# Carbon Paper Modified with Functionalized Poly(diallyldimethylammonium chloride) Graphene and Gold Phytonanoparticles as a Promising Sensing Material: Characterization and Electroanalysis of Ponceau 4R in Food Samples

**DOI:** 10.3390/nano12234197

**Published:** 2022-11-25

**Authors:** Natalia Yu. Stozhko, Ekaterina I. Khamzina, Maria A. Bukharinova, Aleksey V. Tarasov, Veronika Yu. Kolotygina, Natalia V. Lakiza, Ekaterina D. Kuznetcova

**Affiliations:** 1 Department of Physics and Chemistry, Ural State University of Economics, 8 Marta St. 62, 620144 Yekaterinburg, Russia; 2 Scientific and Innovation Center of Sensor Technologies, Ural State University of Economics, 8 Marta St. 62, 620144 Yekaterinburg, Russia; 3 Institute of Natural Sciences and Mathematics, Ural Federal University, 620002 Ekaterinburg, Russia

**Keywords:** poly(diallyldimethylammonium chloride), functionalized graphene, «green» synthesis, gold phytonanoparticles, nanomodifier, carbon paper/veil, sensing material, electrochemical sensor, Ponceau 4R, food dye, electrosensing, soft drinks, candies, popsicles

## Abstract

This paper presents a novel eco-friendly sensing material based on carbon paper (CP) volumetrically modified with a composite nanomodifier that includes functionalized poly(diallyldimethylammonium chloride) graphene (PDDA-G) and phytosynthesized gold nanoparticles (phyto-AuNPs). The functionalization of graphene was justified by Fourier-transform infrared spectroscopy. The phyto-AuNPs (d = 6 nm) were prepared by “green” synthesis with the use of strawberry leaf extract. The sensing material was characterized using scanning electron microscopy, electrochemical impedance spectroscopy, and voltammetry. The research results indicated a more than double increase in the electroactive surface area; a decrease in the resistance of electron transfer on nanocomposite-modified CP, compared to bare CP. The phyto-AuNPs/PDDA-G/CP was used for the electrosensing of the synthetic dye Ponceau 4R. The oxidation signal of colorant enhanced 4-fold on phyto-AuNPs/PDDA-G/CP in comparison to CP. The effect of the quantity of nanomodifier, solution pH, potential scan rate, accumulation parameters, and differential pulse parameters on the peak current of Ponceau 4R was studied. Under optimal conditions, excellent sensory characteristics were established: LOD 0.6 nM and LR 0.001–2 μM for Ponceau 4R. High selectivity and sensitivity enable the use of the sensor for analyzing the content of Ponceau 4R in food products (soft drinks, candies, and popsicles) without additional sample preparation.

## 1. Introduction

Dyes are one of the most popular food additives along with preservatives, stabilizers, flavorings, and aroma enhancers. They aim to give, enhance, or restore the color of food products. Synthetic dyes have a wide range of shades, give products a color that is stable over time, and have high water solubility and chemical inertness. Their production is relatively cheap, which economically benefits manufacturers. Synthetic dyes are derived from chemical compounds such as benzene, toluene, and aniline substances released from coal tar through a series of chemical reactions such as sulfonation, nitrification, and nitration.

When used excessively, synthetic dyes, especially those containing aromatic rings and azofunctional groups, become potentially toxic to the human body [1,2]. Decomposition products can accumulate and provoke allergic reactions, the development of cancer, and disorders of the gastrointestinal tract, respiratory tract, and central nervous system [3]. The harmful effect of the dye largely depends on its concentration in the product, which is why the content of dyes in food products is regulated by the European Food Safety Authority (EFSA, Parma, Italy). Ponceau 4R (E124), a synthetic azo dye with an attractive bright red color, is widely used in the food industry for coloring soft drinks, syrups (including pharmaceutical syrups), sauces, flavorings, jellies, and sweets. According to the EFSA requirements [4], the permissible level of Ponceau 4R (E124) in soft drinks should not exceed 100 mg L^−1^.

Several analytical methods have been developed for the routine detection of Ponceau 4R, including spectrophotometry, chromatography (HPLC), and capillary electrophoresis [5]. Along with the advantages, each of the methods has some disadvantages. The spectrophotometric method is one of the most commonly used methods of analysis. However, its use in the determination of dyes is limited due to the lack of absorption specificity, caused by the overlap of spectral bands in dye mixtures [6]. Chromatographic methods for dye separation, for example, HPLC, are quite effective if the stationary phase is chosen correctly, but it should be noted that the analysis is time-consuming and organic reagents are abundantly used as mobile phases [7]. Capillary electrophoresis, despite its high efficiency in dye separation, has a worse reproducibility than chromatography. In addition, the method efficiency is strongly dependent on the temperature factor.

**Figure 1:**
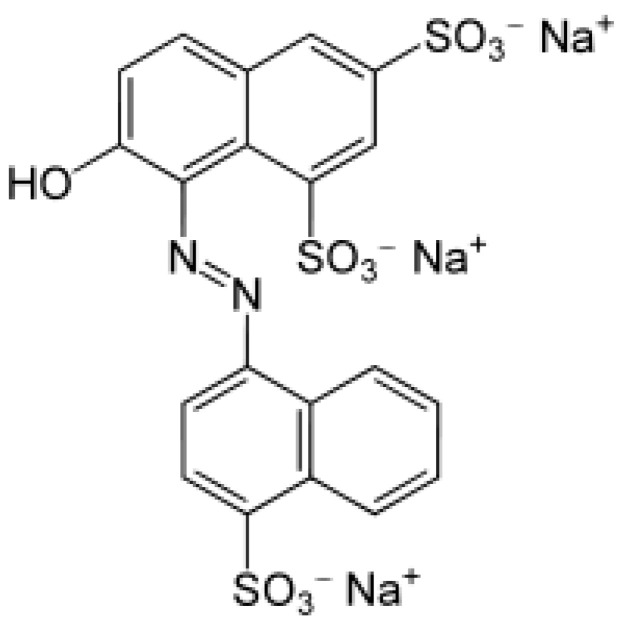
Structural formula of Ponceau 4R (E124).

Compared to the aforementioned methods, electrochemical methods demonstrate a simple, fast, highly sensitive, and selective determination of food contaminants using inexpensive, portable equipment that allows for in situ analysis. The presence of aromatic (OH–) and azo (–N=N–) groups in the structure of Ponceau 4R ensures its ability to be oxidized and reduced (Figure 1). The azo group can participate both in oxidation processes with the formation of radicals and a polymerized azo fragment, and in the reduction to an aromatic amino group or hydrazo group. Its oxidation on the surface of a solid indifferent substrate proceeds more easily than the oxidation of the hydroxyl group. For analytical purposes, and in the identification of Ponceau 4R, an anodic signal that occurs during its electrochemical oxidation is most widely used.

When determining Ponceau 4R by applying anodic voltammetry, in most cases, sensors based on modified glassy carbon and carbon paste electrodes are used. Various carbon materials are widely used as modifiers to increase the surface area and the sensor’s electrical conductivity, namely: reduced graphene oxide [8,9], cyclodextrin-functionalized graphene aerogels [10], multi-walled [11,12] and single-walled [13] carbon nanotubes, as well as porous carbon obtained as a result of the carbonization of CaCO_3_ nanoparticle composite at 800 °C as a solid template and starch as a precursor (starch/nano-CaCO_3_ ratio 1:1) [14]. The Ponceau 4R analytical signal is positively affected by electrode modification with alumina microfibers [15] and ԑ-MnO_2_ microspheres with a uniform flaky texture, which exhibit excellent electrocatalytic properties [16]. The synergistic effect, resulting from the presence of diamond nanoparticles and WS_2_ nanosheets in the sensitive layer of the sensor, contributed to the detection of Ponceau 4R at a lower potential of +0.2 V than the potential of +0.69 V in their absence [17]. An increase in the Ponceau 4R oxidation current and a decrease in its potential were observed on a biopolymer [poly(L-Cysteine)]-modified glassy carbon electrode [18].

High electrocatalytic activity with regard to Ponceau 4R oxidation was demonstrated by an electrode based on a composite containing solid paraffin and an ionic liquid-modified expanded graphite paste electrode [1]. In comparison with unmodified electrodes, a 7.5-fold increase in the peak oxidation current of Ponceau 4R was observed on the ionic liquid-modified expanded graphite paste electrode, which can be explained by the sensitizing effect of the ionic liquid. This effect may be associated with higher ionic conductivity and easier electron transfer.

Along with ionic liquids, researchers focus on metal-organic frameworks (MOFs). The latter have crystalline networks obtained by binding a coordination center—a metal ion—to polyfunctional organic molecules. MOFs are porous materials that greatly increase the effective surface area of the electrode. Darabi R. et al. [19] described a carbon paste electrode modified with 1-ethyl-3-methylimidazolium chloride and Cu-BTC MOF. Their findings showed that the presence of both Cu-BTC MOF and ionic liquid causes a significant increase in the peak oxidation current of the dye. This could also be a result of adsorption processes. The negative shift of the Ponceau 4R oxidation potential on Me-MOF (Me: Ni, Co, Zn)-modified electrodes indicates the catalytic ability of the frameworks [20]. The Ni-MOF-modified glassy carbon electrode, which has the best electrocatalytic activity among Me-MOF electrodes, showed the strongest electrochemical response towards redox probes and Ponceau 4R in solutions.

One of the latest reviews, describing the achievements in the field of the electroanalysis of Ponceau 4R [21], reports that, due to the combination of various characteristics, modified electrodes based on carbon nanomaterials are the most effective for Ponceau 4R monitoring. In this regard, important and promising are the studies aimed at finding new carbon-containing materials and their composites that would be environmentally friendly, cheap, and affordable, all while able to ensure the high analytical and operational characteristics of both the sensor and the analysis at the sampling site. Recently, carbon paper/carbon veil has been attracting widespread research interest, due to its high level of electrical conductivity, large surface area, and 3D bulk structure, which ensures the strong fixation of the modifier in the carbon fiber network. Carbon paper and carbon veil are two different names for almost the same material. In this article, we will use the term carbon paper (CP). Other important advantages of this material are the low cost and the suitability for the mass production of planar sensors, which, together with portable equipment, are capable of performing *on-site* and *in-site* analysis. So far, a few CP-based electrochemical sensors have been developed. They are described in the review [22] and some original articles that have not been included in this review [23,24,25]. Quite recently, the first article has been published that presents a novel voltammetric sensor based on carbon paper modified by graphite powder for the highly sensitive and selective determination of sunset yellow and tartrazine [26]. This sensor was used for analyzing non-alcoholic and alcoholic drinks. The study showed highly reproducible and correct results. However, as the literature shows, CP-based sensors for Ponceau 4R monitoring have not been developed yet.

When working on a new CP sensor for the determination of Ponceau 4R, it is reasonable to modify the carbon fiber matrix of carbon-supported nanomaterials, in order to obtain better electrical contact between the fibers and the transducer, a larger active surface area, and a lower overvoltage. Graphene is an attractive nanomaterial for this task. It is a two-dimensional crystal with high charge carrier mobility, good electrical conductivity, mechanical strength, and high electrocatalytic activity. However, the aggregation and repacking of graphene nanosheets due to their strong π-π interaction creates difficulties in obtaining reproducible properties in this nanomaterial. A promising strategy for separating graphene nanosheets, increasing their hydrophilicity, improving the stability of their suspension, and increasing their electrical conductivity is the functionalization of the graphene surface with conductive polymers. Poly(diallyldimethylammonium chloride) (PDDA) is a cationic polymer electrolyte capable of effectively stabilizing graphene by changing its electrostatic charge and weakening the van der Waals’ and π-π interactions between graphene nanosheets, and enhancing the hydrophilicity and exfoliation of graphene nanosheets in aqueous media [27]. PDDA is prepared by cyclic radical polymerization in an aqueous medium in the presence of an initiator, e.g., ammonium persulfate [28]. Graphene-functionalized PDDA (PDDA-G) has good conductivity, solubility, and biocompatibility, and can be easily decorated with other nanomaterials to impart new properties to the material in order to use it in electrochemical sensors [29,30,31,32,33]. Thus, to determine trace quantities of ferulic acid, a glassy carbon electrode (GCE) modified with PDDA-G, i.e., PDDA-Gr/GCE [29], was used; to determine N-nitrosodiphenylamine, PDDA-G/PtNPs/GCE (where PtNPs are platinum nanoparticles) [30]; to determine puerarin, CdTe-PDDA-G/GCE (where CdTe are quantum dots); to determine dopamine, PDDA-G/AuNPs/GCE (where AuNPs are gold nanoparticles) [31]; to determine allura red, PDDA-G/NiNPs/GCE (where NiNPs are nickel nanoparticles) [32]; and to determine quinoline yellow, PDDA-RGO (where RGO is reduced graphene oxide) [33]. The positively charged surface of functionalized PDDA-G favors interaction with anionic forms of analytes. The presence of sulfonate anionic groups, naphthalene aromatic rings, and an azo fragment in the structure of Ponceau 4R should facilitate interaction with PDDA-G cationic centers, thus ensuring sufficient accumulation on the modified electrode surface.

The unique characteristics of gold nanoparticles such as high surface energy, a high surface-to-volume ratio, chemical stability, high catalytic activity, and their ability to function as electron transport mediators, have ensured their wide use in electrochemical sensors [34,35]. It has been established that gold nanoparticles, synthesized using a strawberry leaf extract with high antioxidant activity, have greater aggregative stability, better electrochemical activity, and provide better analytical characteristics for the determination of ascorbic and uric acids, compared to citrate gold nanoparticles synthesized by the Turkevich method [24,36]. By additionally decorating the PDDA-G material with phytosynthesized gold nanoparticles, one would expect an improvement in the electron transfer and electrocatalytic properties of the sensor.

Since almost no evidence is available in the literature with regard to carbon paper modified with functionalized poly(diallyldimethylammonium chloride) graphene and gold phytonanoparticles, there is a need to examine this promising sensory material.

The aim of this study is: to develop a new sensing material from carbon paper modified with poly(diallyldimethylammonium chloride)-functionalized graphene/phyto-gold nanocomposite; to characterize this material; and to use it for the determination of Ponceau 4R in foodstuffs, including soft drinks, candies, and popsicles.

## 2. Methods and Materials

### 2.1. Chemicals and Reagents

For this study, the following materials were acquired; carbon veil/paper with a 20 gm^2^ surface density (Jiaxing Fu-Tech New Materials Co., Ltd., Jiaxing, China); polyethylene terephthalate film (Fellowes Inc., Itasca, IL, USA); graphene oxide (Rusgraphen Ltd., Moscow, Russia); diallyldimethylammonium chloride (DDA) (Sigma Aldrich, Saint Louis, MO, USA); hydrazine (JSC Reactiv, St. Petersburg, Russia); HAuCl_4_ (RPE Tom’analit Ltd., Tomsk, Russia); Na_2_HPO_4_·12H_2_O and (NH_4_)_2_S_2_O_8_ (JSC Vekton, St. Petersburg, Russia); KH_2_PO_4_ and HCl (NevaReaktiv Ltd., St. Petersburg, Russia); K_3_[Fe(CN)_6_)] (AO Reachim Ltd., Moscow, Russia); KCl and NaOH (JSC ChemReactivSnab, Ufa, Russia); Ponceau 4R (Merck KGaA, Darmstadt, Germany); taurine and ascorbic acid (Sigma-Aldrich Co, St. Louis, MO, USA); glucose (NevaReaktiv Ltd., St. Petersburg, Russia); tartaric acid (Merck KGaA, Darmstadt, Germany); sodium citrate, ammonium chloride and sodium carbonate (JSC ChemReactivSnab, Ufa, Russia); and Blue Ribbon ashless filter paper (Melkhior XXI Ltd., Reutov, Moskovskaya Oblast, Russia).

All reagents were of chemical purity and used without extra purification. Solutions were prepared by dissolution in deionized water.

### 2.2. Instruments

CP electrodes were fabricated using an LM-260iD laminator (Rayson Electrical MFG, Ltd., Foshan, China). A capillary glass viscometer VPZh-2m with an internal capillary diameter of 0.56 mm (Labtekh Ltd., Moscow, Russia) was used to determine the molecular weight of PDDA.

Microscopic studies and energy-dispersive analysis of the sensing material were carried out using a Zeiss EVO MA 15 scanning electron microscope (Carl Zeiss Industrielle Messtechnik GmbH, Oberkochen, Germany) equipped with an X-Max^N^ 80 SDD energy dispersive detector (Oxford Instruments Analytical Ltd., High Wycombe, UK). An accelerating voltage of 20 kV was used to obtain SEM images. Electrochemical measurements were carried out on µAutolab Type III potentiostat/galvanostat (Metrohm, Herisau, Switzerland) with a three-electrode cell. The cell included a bare/modified CP electrode as a working electrode, a silver chloride reference electrode EVL-1M3.1 (Ag/AgCl/KCl, 3.5 M) (JSC Gomel Plant of Measuring Devices, Gomel, Belarus), and a carbon rod as an auxiliary electrode. Fourier-transform infrared spectroscopic studies were carried out using an Agilent Cary 6300 FTIR spectrometer with a spectrum resolution of <2 cm^−1^ and equipped with an attenuated total reflection attachment (Agilent Technologies Inc., Durham, NC, USA).

The pH of the background electrolyte was monitored using a pH/ions-meter TA-Ion (RPE Tomanalyt Ltd., Tomsk, Russia). The dispersion of graphene oxide (GO) was carried out using an ultrasonic bath RH PS-40A (Shenzhen Codyson Electrical Co., Ltd., Shenzhen, China). The magnetic heated stirrer RCT basic (IKA-Werke, Staufen, Germany) was used to synthesize PDDA-G and phyto-AuNPs. An Akvalab-UVOI-MF-1812 installation (JSC RPC Mediana-Filter, Moscow, Russia) was used to obtain deionized water.

### 2.3. Synthetic Procedures

#### 2.3.1. PDDA Synthesis

PDDA was synthesized with the use of radical polymerization in an aqueous medium [37]. The initial reagent was an aqueous solution of DDA C = 65%. To obtain 3 mL of PDDA, 2.4 mL of the DDA solution was taken. Separately, an aqueous solution of ammonium persulfate (APS) initiating agent (NH_4_)_2_S_2_O_8_, C(APS) = 50 mM was prepared. Subsequently, 0.3 g of PSA was added to the DDA solution, and then the reaction mixture was adjusted to 3 mL with water. The concentration of DDA in the reaction mixture was 55%. Polymerization was carried out at 90 °C for 3 h. The resulting viscous PDDA solution was diluted to 20%.

The molecular weight (M) was determined by viscometry by measuring the viscosity (η) of aqueous solutions. The measurements were taken at 26 °C in 1 M of NaCl solution, compensating for the charge of the macromolecule. To calculate the molecular weight of PDDA, the Kuhn-Mark-Houwink Equation (Equation (1)) was used:

(1) $$\left[\eta \right]=K\cdot M^{a}$$

where K and a are constants for the polymer–solvent system at a given temperature and are equal to 1.12 × 10^−4^ and 0.82 [38].

The molecular weight of PDDA, determined by viscometry, was 15,600 g mol^−1^.

#### 2.3.2. PDDA-G Synthesis

To synthesize PDDA-G, 5 mg of GO was dispersed in 5 mL of deionized water to obtain GO dispersion with 1 mg mL^−1^ concentration. The resulting dispersion was sonicated for 15 min. Next, 100 µL of 20% PDDA was added and mixed for 30 min. Then, 100 μL of hydrazine hydrate was added and kept in a water bath at 90 °C for 5 h. Thereafter, the resulting suspension was centrifuged at 13,000 rpm for 5 min and washed three times with water. The pellet was re-suspended in the original volume of water.

#### 2.3.3. Phyto-AuNPs Synthesis

Gold phytonanoparticles were synthesized in accordance with the procedure proposed in [39]. The strawberry leaf extract with pH 11 was prepared as described in [36] and then used to reduce Au(III) ions to Au(0). The synthesis procedure was as follows: first, 1 mL of an alkaline plant extract was added to 5 mL of 1 mM HAuCl_4_ boiling solution and was constantly stirred with a magnetic heated stirrer. A few minutes later, the color of the solution in the reaction beaker changed from pale yellow to wine red, which is a visual confirmation of the formation of gold phytonanoparticles. The sol was cooled to room temperature, washed twice with distilled water, and centrifuged at 14,000 rpm for 10 min. The washed sol was stored in the refrigerator.

#### 2.3.4. Preparation of Phyto-AuNPs/PDDA-G

**Figure 2:**
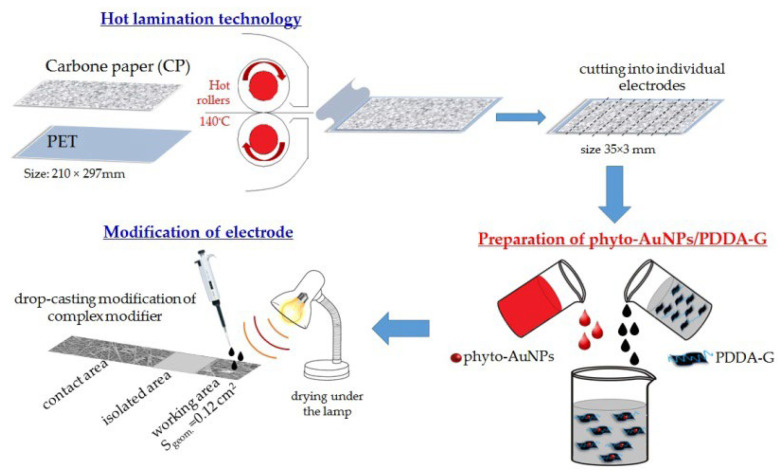
Fabrication of modified carbon paper electrodes.

To prepare phyto-AuNPs/PDDA-G, the phytosynthesized gold sol was added to the PDDA-G suspension and was carefully stirred for 5 min (Figure 2). In order to select the best mixture, different mass fractions of phyto-AuNPs were used in the composition material.

### 2.4. Fabrication of Electrodes

To fabricate electrodes the hot lamination method was applied as described in [24]. Figure 2 illustrates how the electrodes were manufactured. As can be seen in Figure 2, carbon paper was firmly immobilized on polyethylene terephthalate film after rolling through the laminator at 140 °C. Individual electrodes were fabricated by cutting carbon paper, immobilized on polymer film, into strips 3 mm wide and 35 mm long. The working area of the electrode was separated from the contact zone by applying the acetone–cementite mixture (*v*:*v* = 1:5) as isolator. The geometric working area of a carbon paper electrode is 12 mm^2^.

The modification of the CP with a modifier was performed using the drop-casting method. Micro-quantities of a specific modifier as a suspension of GO, phyto-AuNPs, PDDA-G, or phyto-AuNPs/PDDA-G were placed onto the CP surface, and lamp-dried. As a result, modified GO/CP, phyto-AuNPs/CP, PDDA-G/CP, or phyto-AuNPs/PDDA-G/CP electrodes were obtained, respectively.

### 2.5. Electrochemical Procedures

#### 2.5.1. Cyclic Voltammetry

To estimate the area of the electroactive surface of the bare and modified CP, cyclic voltammograms were recorded in a K_3_[Fe(CN)_6_] solution in the potential range of −0.4 V to 1.2 V at a scan rate of 50 mV s^−1^.

When examining the electrochemical behavior of Ponceau 4R on various electrodes, cyclic voltammograms were recorded in the phosphate buffer solution in the potential range of 0.2 V to 1.1 V at a potential scan rate of 50 mV s^−1^.

To study the mechanism of the Ponceau 4R electrochemical transformation, cyclic voltammograms were recorded in the phosphate buffer solution in the potential range of 0.2 V to 1.1 V at potential scan rates ranging from 25 mV s^−1^ to 300 mV s^−1^.

#### 2.5.2. Linear Sweep and Voltammetry

When choosing the best composition and quantity of the modifier, as well as examining the effect of the background electrolyte pH (phosphate buffer solution), linear sweep voltammograms from 0.2 V to 1.1 V and a scan rate of 50 mV s^−1^ were used.

#### 2.5.3. Differential-Pulse Voltammetry

Differential-pulse voltammograms of various Ponceau 4R concentrations were recorded in the potential range of 0.2 V to 1.1 V under the selected optimal conditions with accumulation: modulation amplitude—60 mV; step potential—15 mV; modulation time—100 ms; E_acc_ = 0 V, t_acc_ = 180 s; and potential scan rate—50 mV s^−1^.

#### 2.5.4. Electrochemical Impedance Spectroscopy (EIS)

EIS measurements were performed in 0.1 M KCl in the presence of 5 mM equimolar mixture K3[Fe(CN)6]/K4[Fe(CN)6] at the working potential of 0.225 V in a range of frequency from 0.1 Hz to 100 kHz.

### 2.6. Real Samples

Drinks (non-alcoholic carbonated water and fruit drinks), chewing candies, and popsicles were purchased from a nearby store (Yekaterinburg, Russia). The samples of the drinks were not subjected to additional sample preparation, the exception was the 100- and 200-fold dilution of the drinks.

An amount of 3.5–10 mg of candies was dissolved in warm deionized water and after final solution was obtained, the liquid was filtered through a Blue Ribbon filter. Popsicles were left to melt at room temperature to a liquid state. Subsequently, 0.05–0.10 mL of either the candy or popsicle solution was placed in an electrochemical cell, containing 9.95–9.90 mL of phosphate buffer solution. The accumulation of the dye was performed at 0 V for 180 s.

### 2.7. Results Processing

All measurements were carried out in five parallel experiments. The results were statistically processed with a confidence level *p* = 0.95 and presented as the mean value ± confidence interval.

Equation (2) was used to calculate the limit of detection (LOD)

(2) LOD=3σ/b

where σ is the standard deviation of the analytical signal for Ponceau 4R with the lowest concentration; b is the slope of the I_pa_ vs. C_(Ponceau 4R)_ dependence.

The addition method was used to measure the content of Ponceau 4R in real samples. To assess the accuracy of the measurement, the Recovery value was used which was calculated following the IUPAC recommendations [40].

## 3. Results

### 3.1. PDDA-G Characteristics

#### FT-IR Spectroscopy

**Figure 3:**
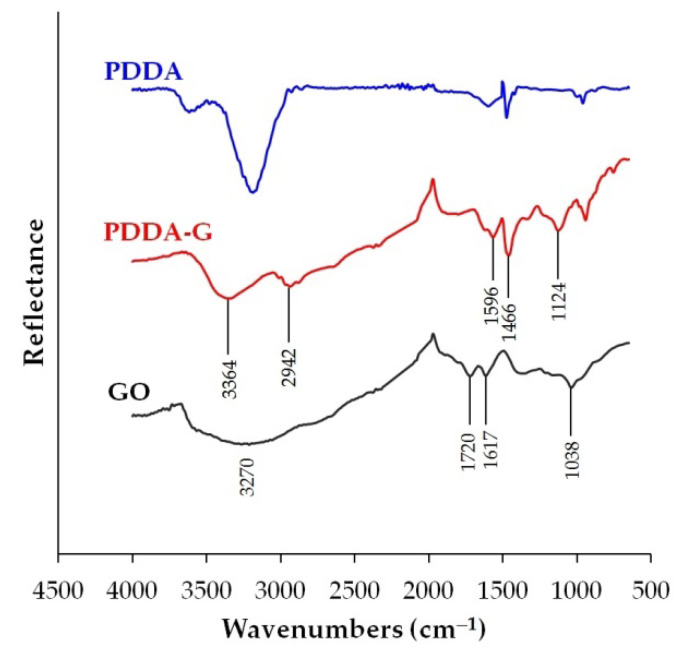
FT-IR spectra of GO, PDDA, and PDDA-G.

Figure 3 presents the FT-IR spectra of GO, PDDA, and PDDA-G. As can be seen in Figure 3, the FT-IR spectrum of GO contains the bands of stretching vibrations of O–H (3270 cm^−1^), C=O (1720 cm^−1^), and C–O (1038 cm^−1^) [41]. These bands are typical and result from the vibrations of surface-oxygenated GO groups. The bands disappear in the PDDA–G system. For the band (1617 cm^−1^) that corresponds to the stretching vibrations of the GO C=C bond, a bathochromic shift by 20 cm^−1^ can be observed. For the PDDA-G system, the bands characteristic of PDDA appear in the FT-IR spectrum, which is caused by the bending vibrations of C–H in the CH_3_ group, bound to the nitrogen atom (1466 cm^−1^), as well as the stretching vibrations of the C–H bond in the CH_2_ and CH_3_ groups (2942 cm^−1^) [29]. A broad band for PDDA-G in the range of 3000–3600 cm^−1^ is the result of the presence of moisture in the sample. The obtained results indicate the fixation of PDDA molecules onto the graphene surface. Additional evidence is the appearance of a new band at 1124 cm^−1^, which can be attributed to the vibrations of the C–N bond formed between graphene and PDDA.

### 3.2. Features of Modified Carbon Paper

#### 3.2.1. Scanning Electron Microscopy (SEM)

**Figure 4:**
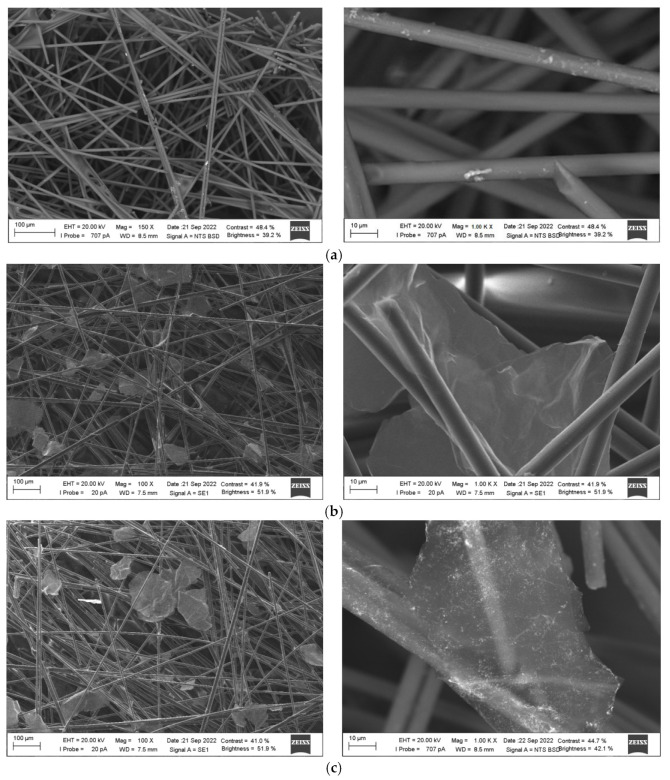
SEM images of bare CP (**a**), CP modified with PDDA-G (**b**) and CP modified with phyto-AuNPs/PDDA-G (**c**).

Figure 4 shows SEM images of the bare CP (Figure 4a), and the CPs with immobilized PDDA-G modifier (Figure 4b) and phyto-AuNPs/PDDA-G (Figure 4c). The bare CP represents randomly woven and sliced polyacrylonitrile carbon fibers with a diameter of 7 µm (Figure 4a). The fibers are connected with a polymer binder, which, filling the space between several fibers, sometimes forms polymer “islands” in non-woven carbon structures. The modification of the carbon paper with flake-shaped, functionalized graphene leads to the filling of many voids between the carbon fibers. As a result, the modified material seems to look denser (Figure 4b). PDDA-G flakes are spread randomly following the arrangement of fibers and are well captured in the ‘web’ structure of the carbon paper (Figure 4b). The introduction of the phyto-AuNPs/PDDA-G nanocomposite also strengthens the contact between the carbon fibers (Figure 4c). As can be seen from Figure 4c, phyto-AuNPs observed as scattered light dots are uniformly distributed predominantly on the translucent surface of PDDA-G. The presence of gold on the PDDA-G nanosheets was confirmed by the results of the EDS analysis (Figure S1).

#### 3.2.2. EIS Measurement

In electrode materials, several factors play an important role in electrotransfer, such as the non-homogeneity of the material composition, multilayeredness and porosity, the non-homogeneous distribution of impurity and defect centers, and the spread in the values of resistance and the capacitance of its microsections. In this case, the ideal circuit elements used in the equivalent circuit method may not be suitable for describing the experimental data. Therefore, to represent real objects, artificial elements are introduced that can take into account the totality of the structural and morphological features of the material and describe its characteristics distributed in space. One of the factors that describes the resistive-capacitive properties of a system in an equivalent electrical circuit is the constant phase element (CPE).

The CPE impedance is expressed by Equation (3) [42]:

(3) $$\mathrm{CPE}={\left[Q{\left(i\omega \right)}^{n}\right]}^{-1}$$

where Q is a pre-exponential frequency independent factor and n is the exponent (−1 ≤ n ≤ 1). Its deviation from 1 characterizes the degree of imperfection of the capacitor. If n = 1, the impedance is ideal capacitance, while it is ideal resistance if n = 0. If 0 < n < 1, this signifies a deviation from the ideal capacitance, which is correlated with surface roughness and defect.

**Figure 5:**
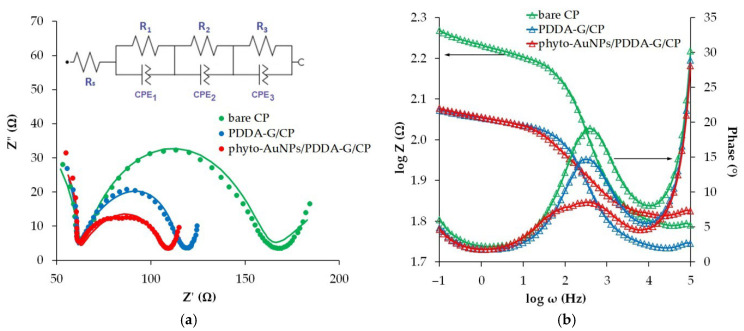
Experimental (points) and fitted (lines) Nyquist plots for bare CP, PDDA-G/CP, and phyto-AuNPs/PDDA-G/CP electrodes in 0.1 M KCl containing 5 mM [Fe(CN)_6_]^3−/4−^. Insert: scheme of equivalent cell (**a**). The Bode and the phase angle plots (**b**).

Taking into account that carbon paper is not homogeneous, as it is composed of highly conductive carbon fibers, the “islands” of acrylic non-conductive binder, and the interface between the contacting fibers, it could be assumed that each local submicroscopic area generates its own combination of RCPE, while the resistive-capacitive pattern observed at the macro level is the sum total of all these contributions. Based on this assumption, the equivalent electrical circuit simulating the behavior of the experimental data was presented as three blocks where the average value of the resistance of electrically conductive carbon fibers is simulated by R_1_; the average value of the resistance of the binder’s areas is simulated by R_2_; and the averaged value of the resistance of the contacting boundaries of the carbon fibers is simulated by R_3_. In order to consider the heterogeneity and non-homogeneity of the heterostructure composition and the spread in the values of the resistance and capacitance of its microsections, a constant phase element was introduced into each block. The inset to Figure 5a illustrates an equivalent electrical circuit simulating the behavior of the experimental data.

The values of the calculated equivalent circuit elements for the unmodified and modified electrodes are presented in Table S1. The data of this table confirm the heterogeneity of the studied electrode structure and the regular improvement in the resistive-capacitive properties when modifying the carbon paper. The small values (from 0.009 to 0.02) of fitting error (χ^2^) indicate the correctness of the equivalent circuit, which takes into account the totality of the structural and morphological features of the material under study.

The good agreement between the experimental and fitted data can also be visually observed on the Nyquist plots. As can be seen from Figure 5a, for bare CP, PDDA-G/CP, and phyto-AuNPs/PDDA-G/CP electrodes in the low-frequency region (0.1–1.5 Hz), the slope of the linear section exceeds 45°, typical for the classical Warburg impedance, which indicates that some additional factors affect diffusion [43]. The reason for this deviation is most likely to be surface roughness, inhomogeneity, and irregularity as well as the porosity of the three-dimensional 3D surface of unmodified and modified carbon paper. All this impedes the diffusion of the electrochemically active component through the 3D material as compared to a homogeneous non-porous flat surface described by the Warburg impedance. As seen in Nyquist plots, the electron transfer resistance, which is displayed as the diameter of a semicircle on the Nyquist diagram, decreases in the series bare CP > PDDA-G/CP > phyto-AuNPs/PDDA-G/CP electrodes. This conclusion is also supported by the Bode plot, which characterizes the value of the logarithm of impedance as the logarithm of frequency (Figure 5b). The modification of the carbon paper leads to a lower height of the impedance jump in the range of medium frequencies (0.1–10 kHz) by a factor of 1.15 for PDDA-G/CP, and of 1.73 for phyto-AuNPs/PDDA-G/CP electrodes when compared with the height of the impedance jump for the CP electrode. Similarly, the maximum phase angle decreases at a frequency of 316 Hz in the transition from unmodified to modified electrodes. Thus, the maximum values of the phase angle are 19°, 15°, and 9° for bare CP, PDDA-G/CP, and phyto-AuNPs/PDDA-G/CP electrodes, respectively. These data confirm the improvement in the conductive (electron-transfer) properties of the modified CP compared to the bare CP.

#### 3.2.3. Evaluation of Electroactive Area

The area of the electroactive surface of the bare and modified CP electrodes was measured with the help of chronoamperograms recorded at E = 0.415 V in 0.1 M KCl containing 1 mM K_4_[Fe(CN)_6_] (Figure S2a) and the Cottrell equation (Equation (4)):

(4) $$I={\mathrm{zFAD}}^{1/2}C/\pi^{1/2}t^{1/2}$$

where I is the anodic peak current; z is the number of electrons transferred (z = 1); F is the Faraday constant (96,500 C mol^−1^); A is the effective surface area of electrode; C is the molar concentration of K_4_[Fe(CN)_6_] (10^−6^ mol cm^−3^); D is the diffusion coefficient (7.6 × 10^−6^ cm^2^ s^−1^); and t is time (s).

Figure S2b depicts the I vs. t^−1/2^ dependences plotted for various electrodes. The slope of the I vs. t^−1/2^ dependences was used to calculate the electroactive area. The calculated area was 18.4 mm^2^ for the bare CP, 21.8 mm^2^ for phyto-AuNPs/CP, 25.7 mm^2^ for PDDA-G/CP, and 31.8 mm^2^ for phyto-AuNPs/PDDA-G/CP. Thus, the electroactive area of phyto-AuNPs/PDDA-G/CP is 1.7 times as large as the electroactive area of the bare CP.

An addition of phyto-AuNPs, PDDA-G, or phyto-AuNPs/ PDDA-G as modifying agents increases the electroactive area of the CP electrode by 18.5%, 39.7%, and 72.8%, respectively. Consequently, the contribution of phyto-AuNPs/PDDA-G to a larger electroactive area exceeds the algebraic sum of the contributions of phyto-AuNPs and PDDA-G, which may suggest the synergetic effect of using a complex nanomodifier.

### 3.3. Ponceau 4R Electrochemical Behavior

**Figure 6:**
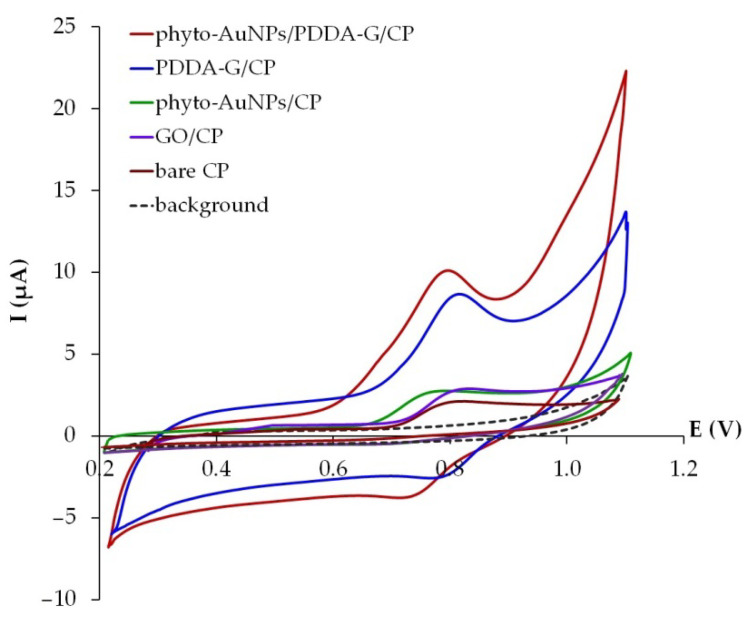
Cyclic voltammograms of 10 μM Ponceau 4R on different electrodes in the phosphate buffer solution (pH 5), ν = 50 mV s^−1^.

The electrochemical behavior of Ponceau 4R was studied using cyclic voltammetry at a scan rate of 50 mV s^−1^. As can be seen in Figure 6, the Ponceau 4R anodic signal, recorded at the potentials of 0.75–0.85 V, grows in the series: bare CP < phyto-AuNPs/CP ≈ GO/CP < PDDA-G/CP < phyto-AuNPs/PDDA-G/CP. An increase in the peak current of the dye oxidation in the presence of the composite modifier components is due to the following factors: an increase in the active surface area upon the introduction of GO into CP; the appearance of active positive charge centers on PDDA-G which allows for the better adsorption of the anionic forms of the dye; and the improved electrocatalytic and conductive properties of the modifier in the presence of phyto-AuNPs.

The cathodic signals of Ponceau 4R on cyclic voltammograms are much weaker and less pronounced than the anodic signals. They are observed on the PDDA-G/CP and phyto-AuNPs/PDDA-G/CP electrodes only. The introduction of phyto-AuNPs (ω = 0.3%) into bare CP and PDDA-G/CP leads to an increase and a shift of 33–35 mV in the anodic signal of the dye towards the cathodic area, which is due to the fact that electrochemical transformation of the dye is fostered by the presence of gold nanoparticles, having electrocatalytic activity. The anodic peak current of Ponceau 4R was used in further studies.

### 3.4. Effect of Modifier Quantity

**Figure 7:**
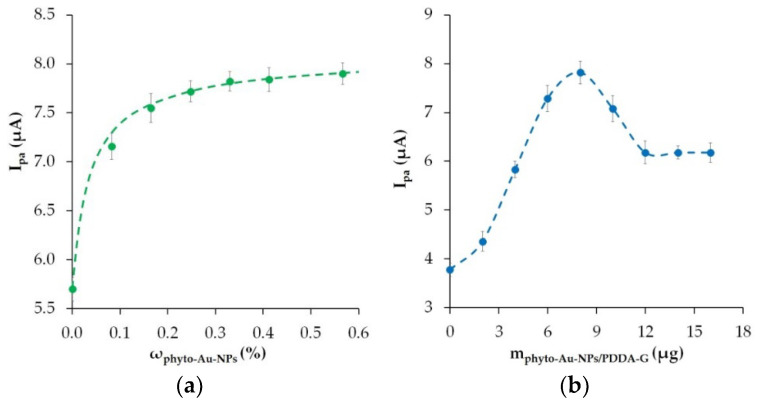
The effects of phyto-AuNP mass content in nanocomposite (**a**) and phyto-AuNPs/PDDA-G quantity in sensing material (**b**) on 10 μM Ponceau 4R signal.

To determine the quantity of the nanocomposite modifier, the dependence of the peak current of the Ponceau 4R oxidation on the mass fraction of phyto-AuNPs in the phyto-AuNPs/PDDA-G modifier was studied. As shown in Figure 7a, an increase in the mass fraction of phyto-AuNPs in the nanocomposite leads to an increase in the anodic peak current of Ponceau 4R. Starting from ω(phyto-AuNPs) = 0.33%, the signal value practically does not change. An increase in phyto-AuNPs in the modifier by more than 0.6% is undesirable, as the Ponceau 4R signal shape can be distorted due to the oxidation current of gold nanoparticles. Thus, the optimal composition of the modifier is 99.67% PDDA-G and 0.33% phyto-AuNPs.

The dependence of the anodic peak current on the weight of the phyto-AuNPs/PDDA-G composite modifier immobilized on CP is illustrated in Figure 7b. The peak oxidation current of Ponceau 4R grows gradually with an increase in the modifier quantity to 8 μg in the sensing material. This is due to the fact that the modifier fills the voids in the CP, thus improving the electron transport properties of the material. With a further increase in the modifier quantity, the anodic peak current goes down. Starting with the amount of 12 μg, the current remains practically unchanged. Apparently, an excess amount (more than 8 μg) of the nanocomposite hinders electron transfer. In further studies, 8 μg of phyto-AuNPs/PDDA-G modifier was applied to the CP electrode.

### 3.5. Effect of pH Value

The impact of the phosphate buffer solution pH on the electrooxidation of Ponceau 4R was studied on the phyto-AuNPs/PDDA-G/CP electrode in the range of 3.0 to 8.0.

**Figure 8:**
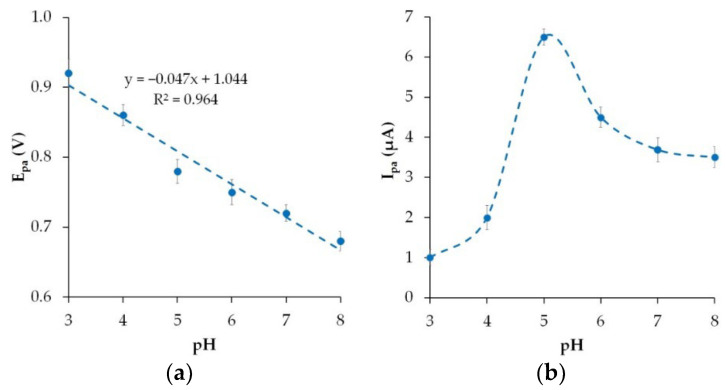
The effects of pH on the anodic peak potentials (**a**) and anodic peak currents (**b**) of 10 μM Ponceau 4R on phyto-AuNPs/PDDA-G/CP electrode.

As can be seen from Figure 8a, the oxidation potential of Ponceau 4R shifts towards the cathodic area as the pH increases, which proves the involvement of protons in this reaction. The E_pa_ vs. pH dependence is linear and has a slope of 47 mV pH^−1^ that is close to the theoretical Nernst value (59 mV pH^−1^). Using Equation (5) and the slope of E_pa_ vs. pH, the ratio of protons (m) vs. electrons (z) involved in the reaction was calculated.

(5) $$E_{\mathrm{pa}}=E^{0}-\frac{59m}{z}\mathrm{pH}$$

The *m*/*z* value of 0.8 indicates an equal number of protons and electrons involved in the electrooxidation of Ponceau 4R. This conclusion is supported by the results of other studies [15]. Since the electrode reaction involves protons, the acidity of the background solution affects the electrochemical behavior of Ponceau 4R. Figure 8b shows the change in the Ponceau 4R peak current as the pH of the phosphate buffer solution changes from 3.0 to 8.0. As the pH changes from 3.0 to 5.0, the anodic peak current grows, peaks at pH 5.0, and then goes down. A decrease in the peak current of the dye at pH > 5.0 is due to the depletion of the medium in the protons directly involved in the electrode process, the competing adsorption between Ponceau 4R and OH^−^ ions, and a change in the ratio of the dye ionic forms present in the phosphate buffer solution at varying pH levels. It was found that Ponceau 4R exists as HR^3−^ in the acidic medium and as R^4−^ in the medium that is closer to neutral and alkaline [44]. Based on the pH effect studies, a phosphate buffer solution with a pH of 5.0 was chosen as the optimal one for further use.

### 3.6. Effect of Potential Scan Rate

**Figure 9:**
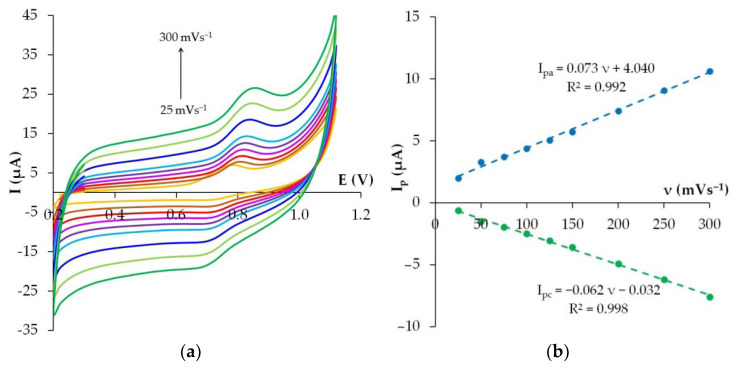
Linear sweep voltammograms of 5 µM Ponceau 4R on phyto-AuNPs/PDDA-G/CP electrode in phosphate buffer solution pH 5 at different potential scan rates: 25, 50, 75, 100, 125, 150, 200, 250, 300 mV s^−1^ (**a**). I_p_ vs. ν (**b**).

To examine the electrochemical transformation process of Ponceau 4R on the phyto-AuNPs/PDDA-G/CP electrode, cyclic voltammograms were recorded at different potential scan rates from 25 to 300 mV s^−1^ (Figure 9a). The cyclic voltammograms show anodic and cathodic peaks of Ponceau 4R separated by 65 mV at a potential scan rate of 50 mV s^−1^, which indicates the quasi-reversible nature of the electrode process, which is also confirmed by other researchers [9].

As can be seen from Figure 9b, the anodic and cathodic peak currents of Ponceau 4R vary linearly with the growing scanning rate in accordance with Equations (6) and (7), which suggests a predominantly controlled adsorption process.

I_pa_ (µA) = 0.073ν (mVs^−1^) + 4.040 (R^2^ = 0.992) (6)

I_pc_ (µA) = −0.062ν (mVs^−1^) − 0.032 (R^2^ = 0.998) (7)

**Figure 10:**
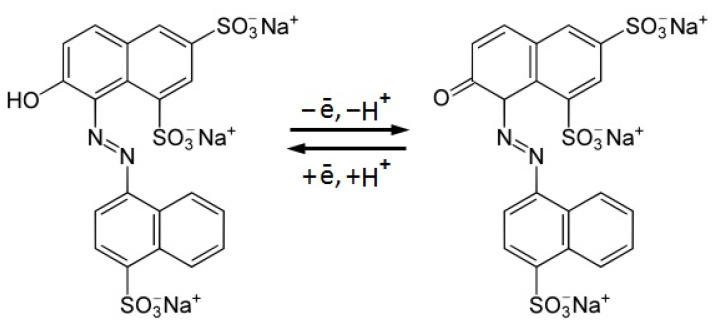
The mechanism of Ponceau 4R electrochemical transformation.

The number of electrons involved in the reversible electrochemical transformation of Ponceau 4R was calculated using Equation (8).

(8) $$\Delta E=\frac{59}{z}$$

where Δ*E* is the potential difference between the anodic and cathodic peak currents of Ponceau 4R taken as 65 mV at υ = 50 mV s^−1^. Therefore, *z* = 0.91, i.e., only one electron is involved in the electrochemical transformation of Ponceau 4R. Based on the data obtained, the proposed mechanism of Ponceau 4R electrochemical transformation is presented in Figure 10.

### 3.7. Effect of Accumulation Parameters

The influence of the potential and the accumulation of Ponceau 4R on the phyto-AuNPs/PDDA-G/CP electrode on the magnitude of the anodic peak current is given in Figure S3. The Ponceau 4R signal grows when the accumulation potential changes from −0.4 to 0.0 V. At a potential of 0.2 V, the Ponceau 4R signal decreases. The peak was recorded at an accumulation potential of 0.0 V. The influence of the accumulation potential on the Ponceau 4R anodic peak current was found at a pH of 5, the pH value for Ponceau 4R existence, mostly as deprotonated R^4−^. The dome-shaped I_pa_ = f (E_acc_) dependence with its maximum at 0.0 V, can be explained by R^4−^ accumulation on positively charged PDDA centers. PDDA bound to graphene is able to acquire a coiled conformation with the high density of a positive charge [45], and ensure R^4−^ accumulation. The shift of the potential from 0.0 V towards the negative area leads to the electrostatic repulsion of R^4−^ from a like-charged surface, and consequently to a decrease in the Ponceau 4R anodic peak current. The shift in the electrode potential from 0.0 V towards the positive area decreases the quantity of the dye concentrated on PDDA, due to the competing influence of the positive charge of carbon paper, which also causes a decrease in the dye signal.

With an increase in the accumulation time from 30 to 180 s, the peak oxidation current of Ponceau 4R grows but it decreases if the accumulation time increases. This type of I_pa_ vs. t_acc_ dependence might be explained by the gradual filling of the active centers of the sensor surface with dye molecules, and then by their partial desorption. The following accumulation parameters—0 V for 180 s—were accepted, taking into account the results of the performed experiments.

### 3.8. Analytical Characteristics of the Phyto-AuNPs/PDDA-G/CP Electrode

#### 3.8.1. Optimization of Experimental Conditions

The optimal conditions for recording the analytical signal of Ponceau 4R on the phyto-AuNPs/PDDA-G/CP electrode in the differential pulse (DP) mode were chosen on the basis of the dependences of the peak current on DP parameters (Figure S4). The strongest Ponceau 4R oxidation signal was obtained under the following conditions:

- A modulation amplitude of 60 mV
- A step potential of 15 mV
- A modulation time of 100 ms

**Figure 11:**
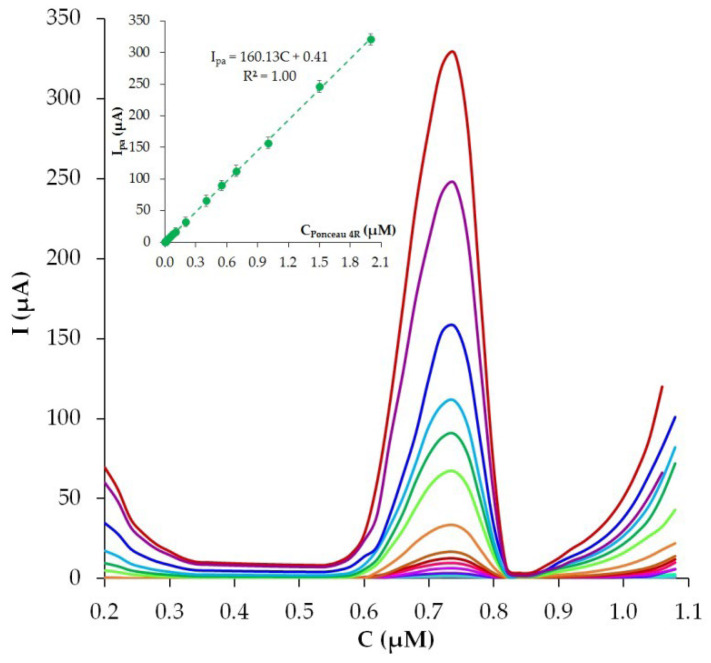
Differential pulse voltammograms of different Ponceau 4R concentrations (0.01–2 µM). Insert: I_pa_ vs. C_(Ponceau 4R)_. Background: phosphate buffer solution pH 5, ν = 50 mV s^−1^, E_acc_ = 0.0 V, t_acc_ = 180 s.

Figure 11 presents differential pulse voltammograms recorded on phyto-AuNPs/PDDA-G/CP under the selected optimal conditions.

The anodic peak current of Ponceau 4R increases with increasing concentration in the range of 1 nM to 2 µM. I_pa_ grows linearly according to Equation (9):

I_pa_ (µA) = 160.13 ± 3.5C_Ponceau 4R_(µM) + 0.41 ± 0.02, R^2^ = 1.0 (9)

The limit of detection (LOD) of Ponceau 4R was calculated at 0.6 nm. The analytical characteristics of the developed phyto-AuNPs/PDDA-G/CP sensor were compared with the characteristics of other sensors (Table 1). It is apparent from Table 1 that phyto-AuNPs/PDDA-G/CP shows a lower detection limit than other sensors, and the linear detection range of Ponceau 4R covers three orders of magnitude, which is comparable to or even better than the existing sensors.

#### 3.8.2. Interference

The influence of various food additives that regulate acidity, sweetness, and preservation was measured against the changes in the 1 μM Ponceau 4R signal in the presence of an interferent. Figure S5 presents the histogram that shows the values of the Ponceau 4R signals in the presence of various interferents relative to the value of the dye signal in the absence of interferents, which was taken as 100%. It was found that the signal does not change significantly (or at the least changes by 5%) in the presence of a 200-fold excess of tartaric acid and a 300-fold excess of ascorbic acid, a 400-fold excess of glucose, a 500-fold excess of taurine, and a 600-fold excess of sodium citrate. In addition, an 800-fold excess of ammonium chloride and a 1000-fold excess of sodium carbonate do not affect the Ponceau 4R signal (Figure S5).

#### 3.8.3. Reproducibility, Repeatability, and Stability

To assess repeatability, the 0.05 µM Ponceau 4R signal was recorded 10 times under the optimal conditions on the same phyto-AuNPs/PDDA-G/CP (Figure S6). The RSD for the given electrode was 2.9%.

The reproducibility was assessed by recording the 0.05 µM Ponceau 4R signal on ten different phyto-AuNPs/PDDA-G/CP electrodes. The electrodes were prepared on different days and the recording was taken on different days by different testers. The RSD obtained for the ten electrodes was 4.8%, which indicates a satisfactory level of reproducibility.

To assess the stability of phyto-AuNPs/PDDA-G/CP, the Ponceau 4R signal was monitored for 4 weeks. Phyto-AuNPs/PDDA-G/CP was stored at 25 °C. The initial Ponceau 4R signal was maintained for one week; by the end of week 4, the signal decreased by 14%.

#### 3.8.4. Real Samples

The developed sensor was used to detect Ponceau 4R in soft drinks, popsicles, and chewing candies purchased from local stores. The analysis was carried out without any additional preparation of the food samples. The obtained data presented in Table 2 show good reproducibility (RSD ≤ 0.052). To confirm the correctness of the results of the analysis, additives of known concentrations were introduced into the samples. The recovery value of 98–102 indicates the accuracy of the results and the applicability of the phyto-AuNPs/PDDA-G/CP sensor for real sample analysis.

## 4. Conclusions

An original sensing material based on carbon paper with graphene-functionalized poly(diallyldimethylammonium) chloride (PDDA-G), and phytosynthesized gold nanoparticles (phyto-AuNPs) was developed. A comprehensive study of the material using FT-IR spectroscopy, scanning electron microscopy, electrochemical impedance spectroscopy, and voltammetric methods made it possible to determine its structure, composition, electron transfer capabilities, and electrochemical properties. It has been found that the impregnation of carbon paper with the phyto-AuNPs/PDDA-G modifier results in an increase in the electroactive surface area, a decrease in the electron transfer resistance, and higher electroactive activity, compared to bare CP. Due to its unique structure and composition, and its good adsorption and electrochemical properties, the developed material has great potential to be employed for electrochemical sensing applications. The phyto-AuNPs/PDDA-G/CP showed enhanced electrochemical activity towards the oxidation of Ponceau 4R. The sensor exhibits a low detection limit of 0.6 nM; a wide range of linearity at 0.001–2 µM; good reproducibility (RSD = 4.8%); repeatability (RSD = 2.9%); and a stable analytical signal for Ponceau 4R. The determination of the dye does not interfere with many interferents that are found in food products. The applicability of the sensor was verified by the determination of Ponceau 4R in soft drinks, chewing candies, and popsicles. The recovery range was between 98 and 102%, which confirms the accuracy of the analysis results.

## Figures and Tables

**Table 1:** Comparison of the analytical performance of phyto-AuNPs/PDDA-G/CP and the existing sensors for Ponceau 4R determination.

Sensor	LOD (nM)	LR (µM)	Method	Accumulation Time/Potential	Reference
Alumina microfibers/CPE	0.8	0.001–0.1	DPV	180 s/0.4 V	[15]
CNT-ppy/GCE	1	0.008–0.1, 0.1–1.0	SWV	300 s	[46]
Poly(EDOT-AA-*co*-EDOT):PSS– SWCNTs–PVA/GCE	1.8	0.0055–110.6	DPV	120 s	[13]
EGPE	2	0.06–4	SWV	400 s/0.4 V	[47]
GAs/GCE β-CD/GAs/GCE	3 0.3	0.01–1.0 0.001–1.0	DPV	180 s/0.2 V	[10]
PC/GCE	3.5	0.004–1.654	DPV	240 s/0.1 V	[14]
TiO_2_/ERGO/GCE	4	0.01–5	AdSDPV	120 s/−0.1 V	[9]
MIPs/Ce_2_Mo_3_O_13_/MWCNTs/GCE	7	0.01–1	DPV	65 s	[48]
r-GO/GCE	28.4	0.2–20	SWV	30 s	[8]
poly(L-Cysteine)/GCE	37.3	1–10	Chronocoulometry	240 s	[18]
WS_2_/DNP/GCE	7800	5–50	DPV	15 s	[17]
phyto-AuNPs/PDDA-G/CP	0.6	0.001–2	DPV	180 s/0 V	This work

GCE—glassy carbon electrode; CPE—carbon paste electrode; CNT—carbon nanotubes; ppy—polypyrrole; poly(EDOT-AA-*co*-EDOT)—poly(acrylate-modified 3,4-ethylenedioxythiophene-co-3,4-ethylenedioxythiophene; PSS—poly(styrene sulfonate SWCNTs—single-walled carbon nanotubes; PVA—poly(vinyl alcohol); EGPE—expanded graphite paste electrode; GAs—three-dimensional porous graphene aerogels; β-CD—β-cyclodextrin; PC—porous carbon; ERGO—electrochemically reduced graphene oxide; MIPs—molecularly imprinted sensor; MWCNTs—multi-walled carbon nanotubes; r-GO—reduced graphene oxide; DNP—diamond nanoparticles; DPV—differential pulse voltammetric; AdSDPV—adsorptive striping differential pulse voltammetric; SWV—square wave voltammetry.

**Table 2:** The results of Ponceau 4R determination in real food samples (n = 5, *p* = 0.95).

Sample	Found in Sample (µM)	RSD (%)	Added (µM)	Found in Sample with Additives (µM)	RSD (%)	Found Additive (µM)	RSD (%)	Recovery (%)
Carbonated soft drink “Barbaris”	83.1 ± 3.1	3.1	85.0	169.8 ± 5.8	1.4	84.8 ± 5.4	2.8	99
Fruit drink	102.1 ± 4.9	4.9	100.0	198.2 ± 2.6	1.1	98.2 ± 2.6	2.1	98
Popsicle	40.8 ± 1.2	1.2	40.0	79.9 ± 3.1	1.5	39.2 ± 3.0	3.1	98
Chewing candy 1	24.4 ± 1.6	5.2	25.0	48.9 ± 2.5	4.1	25.2 ± 0.9	2.9	102
Chewing candy 2	20.4 ± 1.1	4.1	20.0	40.1 ± 1.8	3.6	19.9 ± 1.5	6.2	99

## Data Availability

Not applicable.

## Supplementary Materials

The following supporting information can be downloaded at: https://www.mdpi.com/article/10.3390/nano12234197/s1, Figure S1: SEM image and EDS spectra of phyto-AuNPs/PDDA-G/CP; Table S1: The values of equivalent circuit elements calculated for unmodified and modified CP electrodes; Figure S2: Chronoamperograms obtained at E = 0.415 V on different electrodes in 0.1 M KCl containing 1.0 mM K_4_[Fe(CN)_6_] (a). Plots of I vs. t^−1/2^ obtained from chronoamperograms on the corresponding electrodes (b); Figure S3: Effect of accumulation parameters: potential at t_acc_ = 60 s (a) and accumulation time at E_acc_ = 0 V (b) on 0.1 µM Ponceau 4R anodic peak current. Background: phosphate buffer solution pH 5; Figure S4: Selection of optimal conditions for 0.1 µM Ponceau 4R oxidation on the phyto-AuNPs/PDDA-G/CP electrode and recording of differential pulse voltammograms: modulation amplitude (a), step potential (b) and modulation time (c). Background: phosphate buffer solution pH 5. E_acc_ = 0 V, t_acc_ = 180 s; Figure S5: Relative response of 1 µM Ponceau 4R in the presence of other interferents; Figure S6: Repeatability test curves of 0.05 µM Ponceau 4R in phosphate buffer solution pH 5 at 50 mV s^−1^.
