# Supplementary material for: Carbon Paper Modified with Functionalized Poly(diallyldimethylammonium chloride) Graphene and Gold Phytonanoparticles as a Promising Sensing Material: Characterization and Electroanalysis of Ponceau 4R in Food Samples
Source: Nanomaterials (Basel). 2022 Nov 25;12(23):4197. doi: 10.3390/nano12234197 (PMC9741185; doi:10.3390/nano12234197)
Supplement: Supplementary file 1 [file nanomaterials-12-04197-s001.zip › nanomaterials-2035387-supplementary.pdf]

Supplementary Materials

Carbon Paper Modified with Functionalized Poly(diallyldimethylammonium chloride) Graphene and Gold Phytonanoparticles as a Promising Sensing Material: Characterization and Electroanalysis of Ponceau 4R in Food Samples

Natalia Yu. Stozhko*, Ekaterina I. Khamzina, Maria A. Bukharinova, Aleksey V. Tarasov, Veronika Yu. Kolotygina, Natalia V. Lakiza, Ekaterina D. Kuznetcova

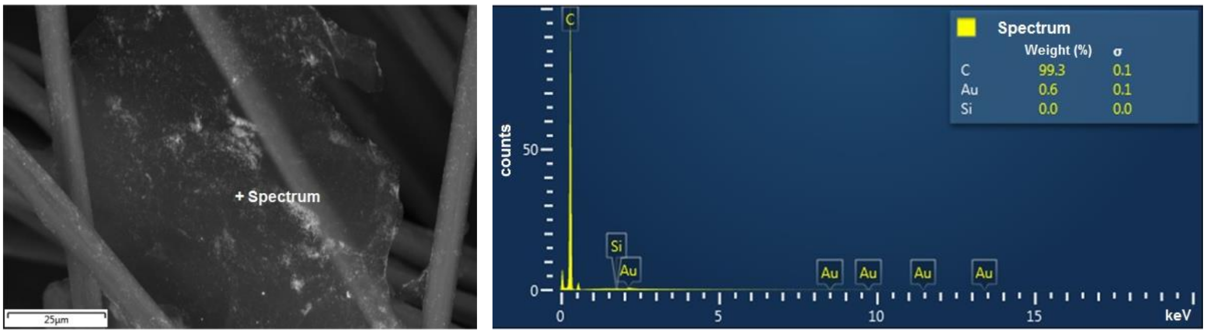

(**a**) (**b**)

Figure S1. SEM image and EDS spectra of phyto-AuNPs/PDDA-G/CP.

Table S1. The values of equivalent circuit elements calculated for unmodified and modified CP electrodes

Electrode	R_1 (Ω)	CPE_1		R_2 (TΩ)	CPE_2		R_3 (Ω)	CPE_3		χ^2
		Q ($n\Omega^{-1}s^{-n}$)	n		Q ($m\Omega^{-1}s^{-n}$)	n		Q ($\mu\Omega^{-1}s^{-n}$)	n	
bare CP	81.2	4.38	1.0	1.1	39.4	0.318	96.6	37.0	0.78	0.01
PDDA-G/CP	55.7	3.38	1.1	1.1	98.3	0.436	55.2	53.9	0.798	0.009
phyto-AuNPs/PDDA-G/CP	54.0	3.88	1.1	1.1	101	0.387	45.4	226	0.666	0.02

As can be seen from Table S1, for CPE_1 (constant phase element), the values are in the range of 1.0–1.1, for CPE_3 – in the range of 0.7–0.8, which is close to 1, i.e., the impedance of these elements and the impedance of the capacitor are identical. For CPE_2, the values of n are in the range of 0.3–0.4, which indicates that the impedance of this element is far from the impedance of an ideal capacitor and represents pseudo capacity. A different order of magnitude of the proportionality factor Q values for all electrodes indicates heterogeneity of their structure.

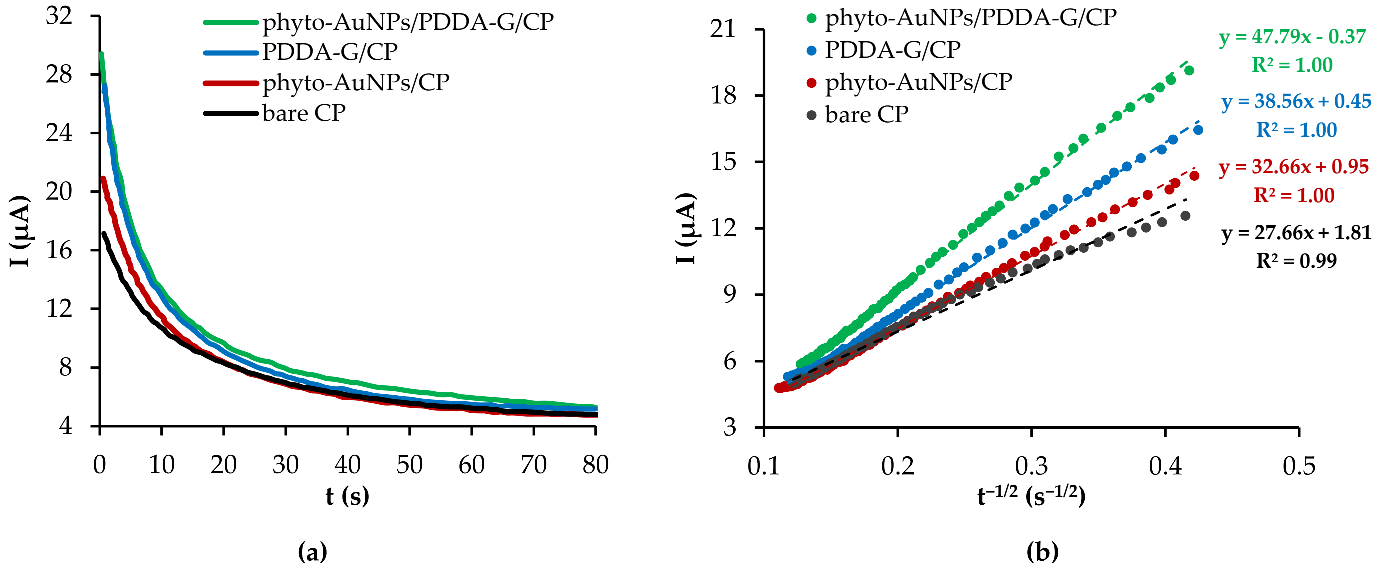

Figure S2. Chronoamperograms obtained at E=0.415 V on different electrodes in 0.1 M KCl containing 1.0 mM $K_4[Fe(CN)_6]$ (**a**). Plots of I *vs* $t^{-1/2}$ obtained from chronoamperograms on the corresponding electrodes (**b**).

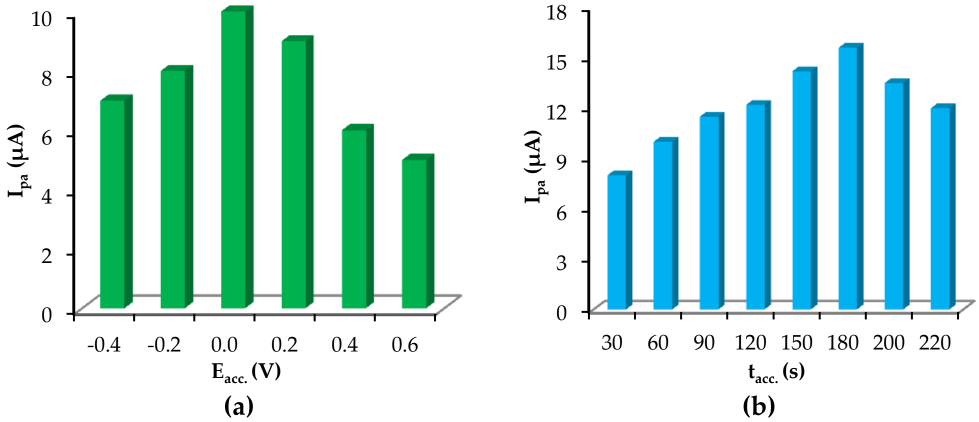

Figure S3. Effect of accumulation parameters: potential at $t_{acc.}$ = 60 s (**a**) and accumulation time at $E_{acc.}$ = 0 V (**b**) on 0.1 µM Ponceau 4R anodic peak current. Background: phosphate buffer solution pH 5.

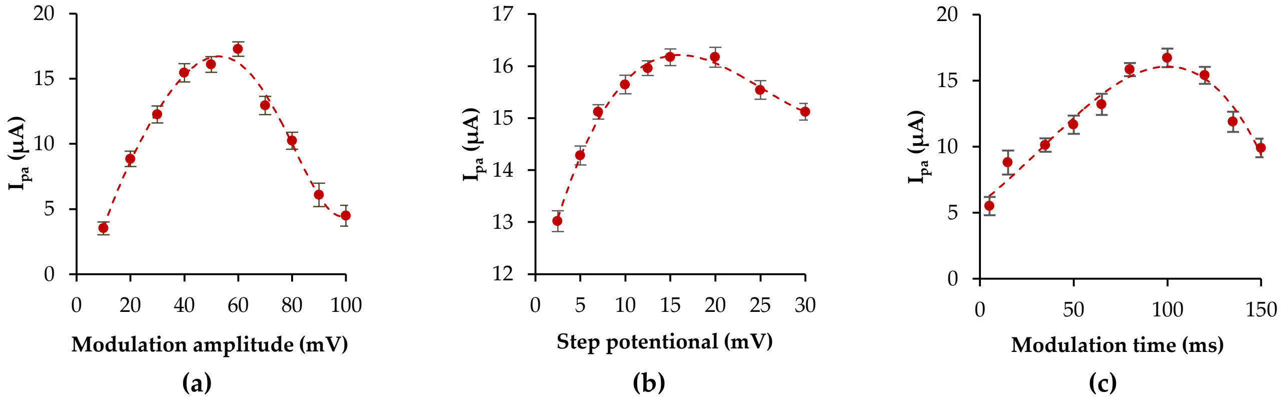

Figure S4. Selection of optimal conditions for 0.1 µM Ponceau 4R oxidation on the phyto-AuNPs/PDDA-G/CP electrode and recording of differential pulse voltammograms: modulation amplitude (**a**), step potential (**b**) and modulation time (**c**). Background: phosphate buffer solution pH 5. $E_{acc.}$=0 V, $t_{acc.}$ = 180 s.

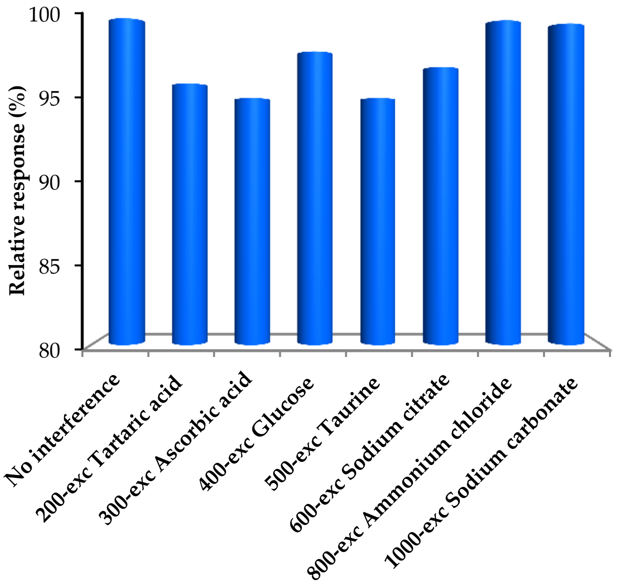

Figure S5. Relative response of 1 μM Ponceau 4R in the presence of other interferents.

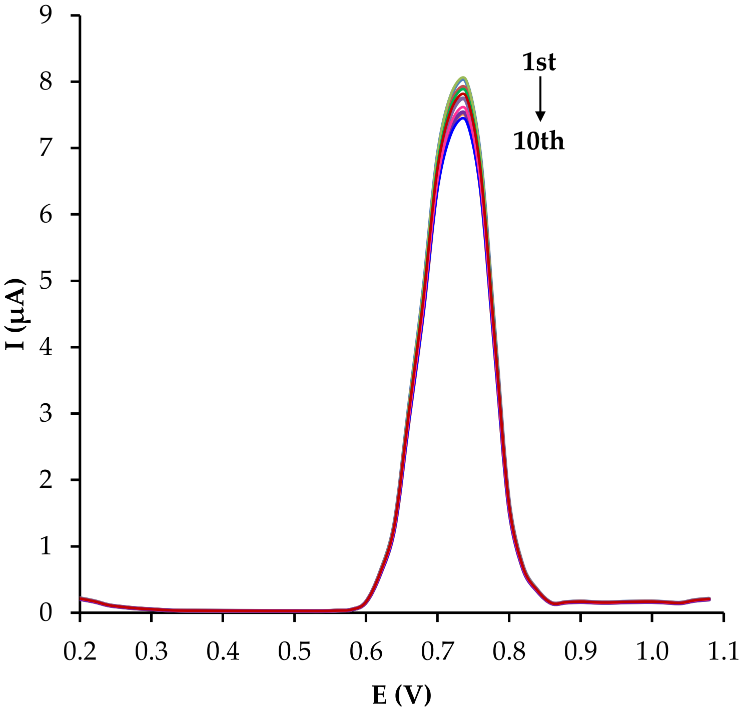

Figure S6. Repeatability test curves of 0.05 μM Ponceau 4R in phosphate buffer solution pH 5 at 50 mV s^{-1}.
